# Examination of Tumor Regression Grading Systems in Breast Cancer Patients Who Received Neoadjuvant Therapy

**DOI:** 10.1007/s12253-020-00867-3

**Published:** 2020-07-20

**Authors:** Anita Sejben, Renáta Kószó, Zsuzsanna Kahán, Gábor Cserni, Tamás Zombori

**Affiliations:** 1 Department of Pathology, Faculty of Medicine, University of Szeged, Állomás u. 1., Szeged, 6725 Hungary; 2 Department of Oncotherapy, University of Szeged, Szeged, Hungary; 3 Department of Pathology, Bács-Kiskun County Teaching Hospital, Kecskemét, Hungary

**Keywords:** Breast cancer, Neoadjuvant therapy, Regression pattern, Grading systems, Residual cancer burden

## Abstract

Neoadjuvant therapy is a common form of treatment in locally advanced breast cancer (LABC) patients. Besides some guidelines for grading regression, a standardized general scheme is not yet available. The aim of our study was to compare the prognostic impact of different regression grading systems, namely the TR/NR, Chevallier, Sataloff, Denkert-Sinn, Miller-Payne, NSABP-B18, Residual Disease in Breast and Nodes and Residual Cancer Burden (RCB) on disease-free (DFS) and overall survival (OS). Data of 746 breast cancer patients treated in neoadjuvant setting between 1999 and 2019 have been included. The different regression grades and follow-up data were collected from medical charts. Statistical analysis included the Kaplan-Meier method, log-rank test and multivariate Cox regression. The average patient age was 55 years. The DFS and OS estimates of patients with complete pathological regression and residual in situ carcinoma have been significantly more favorable than those having partial regression or no signs of regression (pDFS<0.001, pOS < 0.001). Significant differences were found between DFS estimates of classes with partial regression and without regression defined by RCB. Concerning DFS estimates, the RCB classification (*p* = 0.019), while regarding OS data the y-stage (*p* = 0.011) and the nodal status (ypN; *p* = 0.045) were significant prognosticators by multivariate Cox regression. Regression grading systems help the evaluation of regression in LABC patients treated with neoadjuvant therapy. Of the several grading systems compared, the RCB classification makes the best distinction between the outcomes of the different classes, therefore we recommend the inclusion of RCB into the histopathological findings.

**Electronic supplementary material:**

The online version of this article (10.1007/s12253-020-00867-3) contains supplementary material, which is available to authorized users.

## Introduction

Treatment of locally advanced breast cancer (LABC) patients has been one of the great challenges of breast oncology for a long time. Patients with such advanced disease benefit from treatment devised by a multidisciplinary team of specialists: oncologists, surgeons, pathologists and radiologists [1].

Neoadjuvant therapy (NAT) has changed the management of LABC, since it can achieve reduction or even complete regression of the primary tumor and its metastases [2, 3]. This downstaging can allow some patients who would have had mastectomy as surgical treatment to be treated with breast conservation [4]. While receiving NAT, patients have to be under constant oncological and radiological follow-up [5]. The effectiveness of NAT completed with surgical and if needed postoperative endocrine treatment seems to be equivalent with adjuvant therapy on the basis of disease-free (DFS) and overall survivals (OS) [6, 7]. Pathological complete regression occurs more frequently in triple negative or HER-2 positive cancers than in ER positive ones [8, 9].

The work-up of surgical specimen after NAT requires the undivided attention of the pathologist. The identification of the primary tumor bed can be challenging because of its resemblance to fibrotic breast tissue. Insertion of metal clips into the tumor and/or specimen mammography can simplify the identification process. Specimen sampling requires adequate radio-pathological correlation [10, 11]. The evaluation of tumor regression after NAT has to be established with full consideration given to radiology, gross morphology and microscopy.

The characterization of regression differs from country to country due to lack of international consensus on definitions. Pathological complete regression (pCR) implies no residual tumor in the surgical specimen, but the meaning is interpreted variously. In some European countries, pCR generally means the absence of in situ or invasive tumor tissue in the specimen. A significant difference in DFS between ypT0ypN0M0 and ypTisypN0M0 was demonstrated by the German and Austrian Breast Groups [12]. The United States Department of Health and Human Services Food and Drug Administration Center for Drug Evaluation and Research and the American Joint Committee on Cancer define pCR as the absence of residual invasive cancer in the surgical specimen [13, 14].

**Fig. 1:**
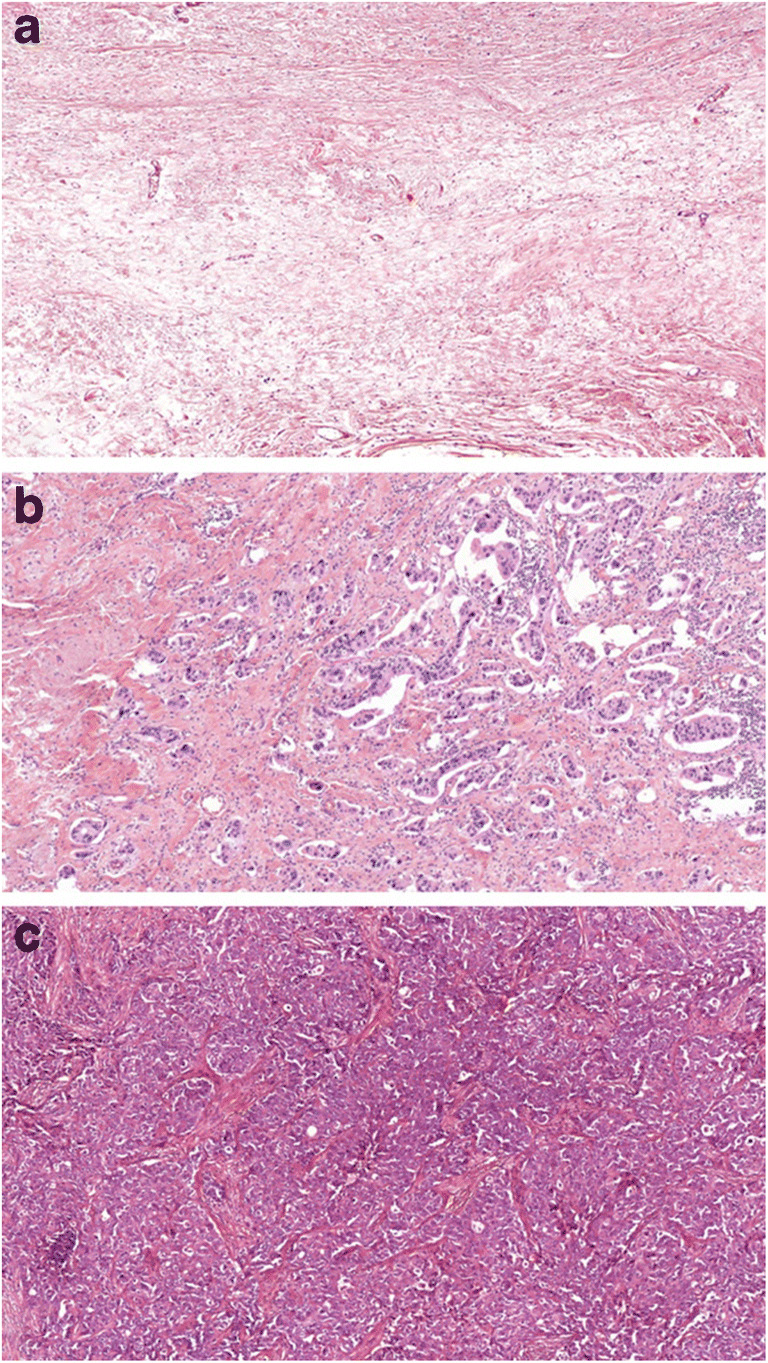
Spectrum of tumor regression: Complete pathological regression (**a**), partial regression (**b**) and lack of regression (**c**) (HE, A: 4x, B and C: 10x)

The histology of post-NAT tumors represents a spectrum from pCR to tumor growth and progression (Fig. 1) [15]. Regression can be reflected by the changes in tumor size, the cellularity of the tumor bed, the presence of lymph node metastases and of ductal carcinoma in situ (DCIS). Since all of these factors may affect prognosis, it is essential that all are represented in the histopathological findings [16]. One of the most essential prognostic factors in breast cancer after NAT continues to be the size of the invasive cancer. In case of unifocal tumors the largest tumor dimension will produce the ypT category, while in cases of multifocal ones the largest diameter of cancer cell containing tissue will be the defining factor.

The evaluation of regression remains a complicated and versatile task especially due to worldwide application of numerous grading systems. The firstly described National Surgical Adjuvant Breast and Bowel Project (NSABP) B18 classifies all NAT cases into two groups. The first group contains pCR cases (including ypT0 and ypTis) whereas the second group refers to all residual invasive tumor cases [17]. Further regression grading systems, namely Chevallier, Sataloff, Miller-Payne, Denkert-Sinn, Residual Cancer Burden (RCB), TR/NR (suggested system in the European guidelines for measuring tumor regression and nodal regression) and Residual disease in breast and nodes (RDBN) define the presence or absence of complete pathological regression with one or more categories for tumors with some regression [18–24]. The TR/NR, Sataloff and RCB systems take residual tumor burden into account, the Chevallier grade considers the presence of some regression, while the Denkert-Sinn grade includes tumor size, and the Miller-Payne system integrates change of cellularity between the biopsy and the resection specimen. The Sataloff, TR/NR and RCB grading systems include lymph node status as well [22, 19, 11]. The RDBN score can be calculated by the following equation RDBN = 0,2xtumor size (mm) + Nottingham histologic grade (1–3) + lymph node involvement (0–3). According to the RDBN score a good (⩽3.4), a moderate (3.4 < and ⩽5.4), and poor (>5.4) prognostic group were identified [24]. The quantification of residual tumor can be performed by using the RCB calculation. The algorithm was developed by Symmans and coworkers and takes notice of the two largest diameters of the residual tumor, the presence and proportion of DCIS and the number of metastatic lymph nodes with the size of the largest nodal metastasis [22]. The evaluation of RCB is supported by the online available RCB calculator (http://www3.mdanderson.org/app/medcalc/index.cfm?pagename=jsconvert3).

Table 1 represents tumor regression grading systems evaluated in our study and defines the differences among them. Although these grading systems are validated, none of them are accepted internationally. The Hungarian protocol in regression grading was recommended by the 3rd Hungarian Consensus Conference on Breast Cancer in 2016 and is practically identical with the recommendation of the European Working Group for Breast Screening Pathology (EWGBSP) [11, 23]. In Germany, the Denkert-Sinn grade is utilized, while in the USA and many other countries the RCB becomes increasingly adopted.

**Table 1:** Tumor regression grading systems for breast cancer specimen after NAT (DCIS: ductal carcinoma in situ, RCB: Residual Cancer Burden, pCR: pathological complete regression, NAT: neoadjuvant therapy)

TR/NR [23]	Chevallier [18]	Sataloff [19]	RCB [22]	Denkert-Sinn [21]	Miller-Payne [20]
TR1a: No residual carcinoma.	G1: Disappearance of all tumor either on macroscopic or microscopic assessment.	TA: Total or nearly total therapeutic effect (i.e. isolated tumor cells).	pCR: ypT0 and ypTis: 0	TRG0: No signs of regression.	G1: No change or some alteration to individual malignant cells but no reduction in overall cellularity.
			(RCB index score)		
TR1b: No residual invasive tumor but DCIS present.	G2: Presence of in situ carcinoma only in the breast, without invasive tumor and tumor cells in the lymph nodes.	TB: Therapeutic effect subjectively superior to 50%.	RCB-I: 0,1–1,35 (RCB index score)	TRG1: Tumor sclerosis with focal inflammation and/or minimal cytopathic changes (>5 mm).	G2: A minor loss of tumor cells but overall cellularity still high; up to 30% loss.
TR2a: Minimal residual disease/near total effect (e.g. < 10% of tumor remaining).	G3: Presence of invasive carcinoma with stromal alteration, such as sclerosis or fibrosis.	TC: Therapeutic effect less than 50%, but evident effect.	RCB-II: 1,36-3,27	TRG2: Great amount of tumor sclerosis. May be multifocal, presence of minimally invasive tumor (Not more than 5 mm, usually with intraductal spread).	G3: Between an estimated 30% and 90% reduction in tumor cells.
			(RCB index score)		
TR2b: Evidence of response to therapy but with 10–50% of tumor remaining.	G4: No or few modifications of the tumoral appearance.	TD: No therapeutic effect.	RCB-III: >3,28	TRG3: No signs of residual invasive tumor.	G4: A marked disappearance of tumor cells such that only small clusters or widely dispersed individual cells remain; more than 90% loss of tumor cells.
			(RCB index score)		
TR2c: > 50% of tumor cellularity remains evident, when compared with the previous core biopsy sample, although some features of response to therapy present.				TRG4: No signs of invasive or in situ tumor.	G5: No malignant cells identifiable in sections from the site of the tumor; only vascular fibroelastotic stroma remains often containing macrophages. However, ductal carcinoma in situ (DCIS) may be present.
TR3: No evidence of response to therapy					
NR1: No evidence of metastatic disease and no evidence of changes in the lymph nodes.		NA: Therapeutic effect, but no metastasis.			
NR2: Metastatic tumor not detected but evidence of response/down-staging, e.g. fibrosis.		NB: No metastasis, no therapeutic effect.			
NR3: Metastatic disease present but also evidence of response, such as nodal fibrosis.		NC: Therapeutic effect, but metastasis.			
NR4: Metastatic disease present with no evidence of response to therapy.		ND: Metastasis, no therapeutic effect.			

The aim of our study was to evaluate the prognostic impact (on disease-free and overall survival) of the different tumor regression grading systems in breast cancer patients treated with NAT. We also aimed to identify which of the grading systems could best reflect prognosis.

## Materials and Methods

NAT receiving, consecutive patients operated on for histologically verified invasive breast carcinoma at the Department of Surgery, University of Szeged or Bács-Kiskun County Teaching Hospital, Kecskemét between 1999 and 2019 were included in our retrospective study. Follow up data were collected from medical charts.

The following clinical and pathological variables were obtained for analysis: age, gender, localization, type of neoadjuvant and surgical treatments, DFS and OS; histological type and grade of cancer in previous core biopsy and surgical specimen, completeness of the resection, vascular invasion, size - possibly in 2 dimensions, ypT, ypN, ystage, tumor cell density, tumor cellularity in biopsy and resection specimens, presence and proportion of DCIS, presence of metastasis and/or regression in lymph nodes, size of metastatic deposits and receptor status (estrogen receptor - ER, progesterone receptor – PR, and human epidermal growth factor receptor-2 – HER2). Tumor cell density was defined as the proportion of viable tumor cells in the complete tumor bed, not including necrosis or DCIS.

Regression grades (NSABP-B18, TR/NR, Chevallier, Sataloff, Denkert-Sinn, Miller-Payne, and RCB) and morphological variables were correlated with DFS and OS data using Kaplan-Meier estimates. Patients were followed from the date of initiation of NAT until the time of recurrence or tumor-related death. Patients alive without recurrence and patients dying from other causes were censored at the time of the last follow-up and death, respectively. The log-rank test was used for pairwise comparisons. All statistical tests were two-sided and *p* < 0.05 values were considered statistically significant. The parameters found significant in the univariable models were entered in multivariable Cox proportional hazard model to identify factors of independent prognostic significance. Statistical models were fitted using SPSS Statistics V.22.0 software (IBM, SSPS 22.0, Armonk, NY USA).

This retrospective study was approved by the regional ethical committee of the Albert Szent-Györgyi Clinical Centre of the University of Szeged.

## Results

Data of 746 patients who underwent NAT and surgical resection were collected. The median patient age was 55 years (range: 26–91) and 2 of them were males. Table 2 summarizes the oncological and surgical treatments of all patients in the examined population. The majority of patients received primary chemotherapies, whereas 16.4% got primary endocrine therapy. Regarding primary systemic chemotherapy, the majority of patients were given third generation (taxane containing) regimens. 11.2% of the patients had been given second generation (anthracycline based) chemotherapeutics. Patients who received a combination of platinum compounds with cyclophospamide fell into the “others” category. Anti-Her2 treatment was essentially given in combination with chemotherapy. Concerning primary endocrine therapy, the most frequent agents used were aromatase inhibitors and the average hormonal therapy treatment period was 1 year. The majority of patients underwent mastectomy. Re-excisions were rarely performed and were done because of positive or close resection margins. Regional lymph nodes were examined in almost all cases, most commonly by means of axillary lymph node dissection.

**Table 2:** Types of NAT and surgical treatment in the examined population (LHRH: Luteinizing hormone-releasing hormone, HER2: Human epidermal growth factor receptor 2, SNB: Sentinel node biopsy, ALND: Axillary lymph node dissection)

Neoadjuvant therapy		
Primary hormonal therapy (*n* = 123 = 100%)	n	%
Tamoxifen	4	3.25
Aromatase inhibitor	102	82.93
Tamoxifen and LHRH-analogue	3	2.44
Aromatase inhibitor and LHRH-analogue	14	11.38
Primary chemo- and target therapy (*n* = 623 = 100%)	n	%
Second generation chemotherapy	70	11.24
Third generation chemotherapy	550	88.28
Others	3	0.48
Anti-HER2 (in combination therapy)	91	14.60
Number of cycles should go under Primary chemo-and target therapy)	5.60	6.00
Surgical treatment (*n* = 746 = 100%)	n	%
Breast conserving excision	249	33.38
Mastectomy	497	66.62
Re-excision	17	2.28
SNB	72	9,65
ALND	593	79,49
SNB + ALND	60	8.04

As Table 3 demonstrates, with histological examination, 87.8% of patients had invasive carcinoma „No Special Type” in surgical specimens. Invasive tubular, mucinous, medullary and metaplastic breast cancers were categorized into the others category. The presence of residual DCIS was described in 212 cases. One fifth of the patients achieved pCR. The most frequent pathological tumor category was ypT2 (20.2%), while 38.9% of the patients fell in with ypN0 category. Most cases expressed ER and PR, while HER-2 positivity was observed in 126 cases (17%). Median patient follow up was 53.8 months (range: 4–238 months; average: 65.1 months). Relapse occured in 34.85% of cases during the follow-up period and tumor specific death was observed in 122 (16,3%) cases.

**Table 3:** Morphological features of breast cancer in the examined population (NST: Invasive carcinoma „No Special Type”, ILC: Invasive lobular carcinoma, DCIS: Ductal carcinoma in situ, R: Resection, V: (Lympho) vascular invasion, Pn: Perineural invasion, HR: Hormone (estrogen and/or progesterone) receptor, HER2: Human epidermal growth factor receptor 2; ypT and ypN categories are defined by AJCC. Not all evaluated features were available for all cases, hence the differences in the sums of some rows

Histological subtype (core)	*n*	%
NST	655	87.80
ILC	55	7.37
others	36	4.83
grade	*n*	%
1	35	4.69
2	246	32.98
3	420	56.30
No data	45	6.03
DCIS (present)	212	28.41
R (R1/R0)	130/616	17.42
V (V1/V0)	151/560	21.23
Pn (Pn1/Pn0)	10/324	2.99
Hormonal state	*n*	%
HR +, HER-2 -	439	58.85
HER-2 +, HR +/-	126	16.89
Triple negative	181	24.26
ypT	*n*	%
ypT0	106	14.21
ypTis	28	3.75
ypT1a	48	6.43
ypT1b	25	3.35
ypT1c	110	14.75
ypT2	151	20.24
ypT3	55	7.37
ypT4	29	3.90
No data	194	26.00
ypN	*n*	%
ypN0	290	38.87
ypN1	227	30.43
ypN2	127	17.02
ypN3	61	8.18
No data	41	5.50
ystage	*n*	%
0	9	1.21
I	75	10.05
II	209	28.02
III	207	27.75
IV	6	0.80
No data	240	32.17

**Fig. 2:**
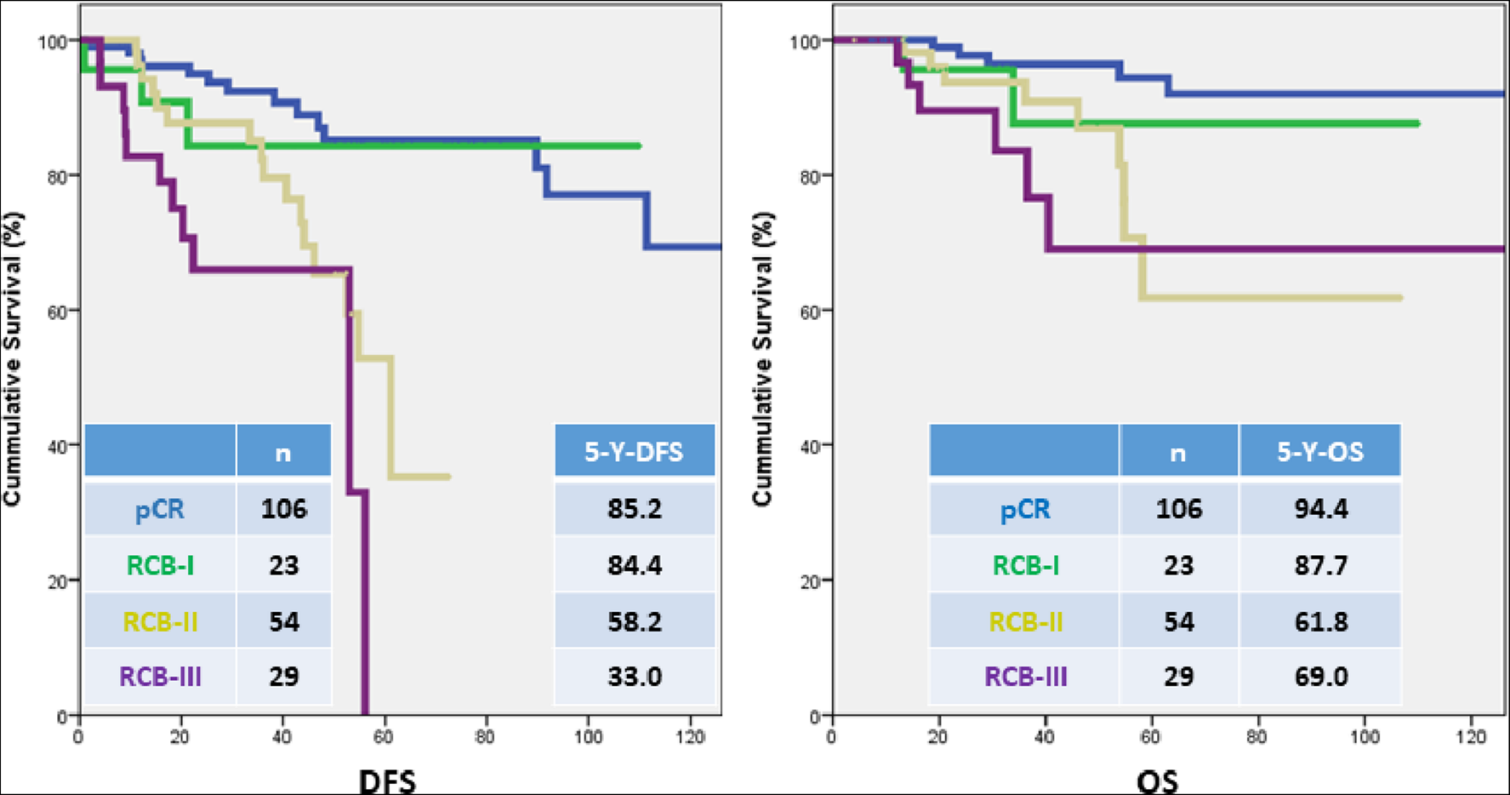
Kaplan-Meier evaluation of the RCB grading system for DFS and OS. Significant differences were found between DFS estimates of pCR vs. RCB-II (*p* < 0.001), pCR vs. RCB-III (p < 0.001), RCB-I vs. RCB-III (*p* = 0.035), RCB-II vs. RCB-III (*p* = 0.05). Regarding OS, significant differences were observed between estimates of pCR vs. RCB-II (*p* = 0.005) and pCR vs. RCB-III (p < 0.001), respectively (RCB: Residual Cancer Burden, DFS: disease-free survival, OS: overall survival, pCR: pathological complete regression)

According to the original histopathology reports, the numbers of patients evaluated with the different regression grading systems are as follows: NSABP-18 grade: 746, Chevallier-grade: 717, Sataloff (T) grade: 494, Miller-Payne grade: 386, TR grade: 392, Denkert-Sinn grade: 348, RDBN grade: 405 and RCB: 212. Figure 2*and* Supplementary Fig. 1-8 show the disease-free survival and overall survival estimates of the different grading systems, respectively. The DFS and OS estimates of complete pathological regression (ypT0) and residual in situ carcinoma (ypTis) together were significantly different from the survivals of tumors without regression and moderate regression categories in all grading systems (*p* < 0.001). There was no significant DFS and OS difference observed between the ypT0 and ypTis categories. Survival values associated with different partial or no response categories showed no significant differences between each other, with the exceptions of DFS for the RCB-I vs III and II vs III categories.

As all regression grading systems showed a significant effect on survival in the univariable models, they were all entered in the multivariable Cox-regression analysis. According to our results the RCB (*p* = 0.019) proved to be an independent prognostic marker for DFS, whereas the ystage (*p* = 0.011) and lymph node status (*p* = 0.045) showed similar results for OS.

## Discussion

Due to the increasing use of NAT in patients having locally advanced breast cancer, more and more articles about its effectiveness have been published [8]. Although imaging techniques serve as great options to monitor regression after NAT, histopathological review remains the gold standard in the evaluation procedure [25]. Although several national guidelines aiming at the standardization of specimen cut up and reporting have been introduced, for example in Australia, Belgium, Germany, the UK, Netherlands, the USA and Hungary, there is no international agreement in the interpretation of tumor regression, in the definition of pCR, and in the measurement of tumor size in cases where fibrosis develops as a result of NAT or multifocality is present [11, 26–31].

Several regression grading systems have been introduced which are based on prognostic markers such as tumor size (in one or more dimensions), change in cellularity, presence of DCIS, presence of regression or metastasis in lymph nodes and the size of lymph node metastasis [17–23]. The definition of pCR and the complete lack of regression -as the extreme ends of the regression spectrum- are common features of these systems which also define one or more subgroups for partial regression categories. Despite of the relative abundance of regression grading systems, there is a lack of international consensus on their application. All grading systems attempt to quantify the degree of regression or the amount of residual tumor, and there is agreement that a quantitative characterization of tumor regression is necessary for the evaluation of the effectiveness of NAT, and may have further role in therapeutic decisions (e.g. alternative treatments if no regression is present).

Although the presence of residual DCIS has been reported to convey a worse prognosis than complete absence of in situ and invasive carcinoma, there was no significant difference between OS and DFS estimates of ypT0ypN0 and ypTisypN0. Our results are therefore supporting the more permissive definition of pCR (including ypTis) defined by the United States’ FDA and endorsed by the AJCC [13, 14] and the European Guidelines [23]. Our findings regarding the prognostic impact of pCR are in keeping with those of others, since patients with pCR had a favorable prognosis (both in DFS and OS) compared to patients having partial regression. Concerning the subcategories of partial regression, we observed significant differences only between DFS estimates of certain RCB classes, namely between RCB-I vs. RCB-III and RCB-II vs. RCB-III classes. No other regression classification system showed subgroups of partial response with significant differences between each other.

The RCB system was developed by Symmans and coworkers in 2007. In their study, the prognostic role of morphological variables was evaluated by Cox-regression, and from variables found statistically significant, a complex equation was produced to determine the RCB index score. The RCB index score was correlated with survival data and cut-off scores were assigned to identify the RCB classes. In concordance with the original results by Symmans et al., there were no significant differences in DFS and OS estimates between RCB-0 (pCR) and RCB-I (nearly pCR) classes. Furthermore, the multivariable Cox regression models for DFS suggest that the RCB system is the only significant prognosticator among regression grades (*p* = 0.019) [22].

In a subsequent publication, Symmans and co-authors have demonstrated that the RCB is a prognostic marker independent from the type of primary chemotherapeutic regime and significant differences have been described between RCB classes among hormone receptor positive (ER+ and/or PR+, HER2-), HER-2 positive (hormone receptor positive or negative) and triple negative (ER-, PR, HER2-) breast cancer cases [32]. Our results support these conclusions, and moreover, by adding primary endocrine therapy to our calculations, RCB remained an independent prognostic marker.

Considering literature data and our results, RCB is highly recommended to be included in routine histopathological reports of breast cancers treated with NAT. Although most elements of RCB are routinely part of histopathological reports, the characterization of some others, namely the second largest dimension of tumor size, the cellularity and the proportion of DCIS, require experience in practice. The standardization of reporting these markers are supported by the concise guidance at the RCB calculator website [32].

Corben and co-authors emphasized the role of the presence and size of lymph node metastasis. Those grading systems that include lymph node status (RCB, Sataloff, TR-NR, RDBN) show better correlation with long term survival than those including only invasive tumor size and cellularity [5]. In keeping with Corben’s results, we found the ypN category as a significant prognostic marker according to OS estimates. The presence of nodal metastasis was associated with poor prognosis regardless of the presence or absence of nodal regression. Corben and co-workers suggested the RDBN grade to be the most optimal regression grading system among the 5 investigated [5]. However, we found no significant differences in DFS or OS between the RDBN groups with Cox regression. This contrast may be due to different factors, like the differences in patients and in cohort sizes (62 vs 746) and the inclusion of primary endocrine therapy in the present analysis.

Concerning the limitations of our study, it has to be mentioned that not all grading systems were assessed in all cases. Several patients had gone through lymphadenectomy prior to NAT and this could influence the prognostic value of a given grading system. Furthermore, the institution where the core needle biopsy was taken differed from the place of surgery in many cases, therefore the comparison of these samples was not always possible. On the other hand, the strengths of our evaluation include a large cohort of patients having primary endocrine treatment or chemotherapy with relatively long follow-up data. Our multicenter study was based on two Hungarian departments with identical cut-up and reporting protocol, following the recommendations of the 3rd Hungarian Consensus Conference on Breast Cancer. Although not all grading systems were evaluated in all cases, even the smallest group included more than 200 patients, and this proved sufficient for statistical analysis.

In our retrospective study involving the grading of response to NAT in 746 patients, we have evaluated and compared the impact of different regression grading systems on DFS and OS. According to our results, the RCB was the best prognostic factor, therefore we would encourage its utilization in routine histopathological reports.

## Electronic Supplementary Material

Supplementary Fig. 1 Kaplan-Meier evaluation of the NSABP-B18 response scheme. Significant differences were defined between DFS and OS estimates of pCR vs. residual invasive tumors (pINV) [p < 0.001] (NSABP: National Surgical Adjuvant Breast and Bowel Project, DFS: disease-free survival, OS: overall survival, pCR: pathological complete regression) (DOCX 160 kb)

Supplementary Fig. 2 Kaplan-Meier evaluation of the Chevallier grading system. Significant differences were observed between DFS estimates of I vs. III group (p < 0.001); I vs. IV group (p < 0.001); II vs. III group (p < 0.001) and II vs. IV group (p < 0.001). Significant distinction was detected between OS estimates of I vs. III group (p < 0.001); I vs. IV group (p < 0.001) and II vs. IV group (p = 0.05) (DFS: disease-free survival, OS: overall survival) (DOCX 217 kb)

Supplementary Fig. 3 Kaplan-Meier evaluation of the Sataloff (T) grading system. Significant differences were seen between DFS estimates of TA vs. TB (p = 0.005), TA vs. TC (p < 0.001) and TA vs. TD (*p* = 0.009) along with significant distinction between OS estimates of TA vs. TB (p = 0.005), TA vs. TC (*p* = 0.016) and TA vs. TD (*p* = 0.003) (DFS: disease-free survival, OS: overall survival) (DOCX 212 kb)

Supplementary Fig. 4 Kaplan-Meier evaluation of the TR grading system. Significant differences were found between DFS and OS estimates of TR1 vs. TR2 (pDFS<0.001; pOS < 0.001) and TR1 vs.TR3 (pDFS<0.001; pOS < 0.001), respectively (DFS: disease-free survival, OS: overall survival) (DOCX 160 kb)

Supplementary Fig. 5 Kaplan-Meier examination of Salatoff (N) and NR grading systems. Significant differences were found between DFS estimates of NR2 vs. NR3 (*p* = 0.027), NR2 vs. NR4 (*p* = 0.020), NR1 vs. NR3 (p < 0.001), NR1 vs. NR4 (p < 0.001) and between OS estimates of NR2 vs. NR4 (*p* = 0.029), NR1 vs. NR3 (p < 0.001) and NR1 vs. NR4 (p < 0.001) (DFS: disease-free survival, OS: overall survival) (DOCX 205 kb)

Supplementary Fig. 6 Kaplan-Meier evaluation of the Denkert-Sinn grading system. Significant differences were defined between DFS estimates of TRG3 vs. TRG0 (*p* = 0.006), TRG3 vs. TRG1 (p = 0.020), TRG3 vs. TRG2 (p = 0.006), TRG4 vs. TRG0 (p < 0.001), TRG4 vs. TRG1 (*p* = 0.012), TRG4 vs TRG2 (p < 0.001) and between OS estimates of TRG4 vs. TRG0 (p < 0.001), TRG4 vs. TRG1 (*p* = 0.038), TRG4 vs. TRG2 (p < 0.001) (DFS: disease-free survival, OS: overall survival) (DOCX 176 kb)

Supplementary Fig. 7 Kaplan-Meier evaluation of the Miller-Payne grading system. The DFS and OS estimates of MP5 group showed significant differences from other groups regarding DFS and OS (p < 0.001) (DFS: disease-free survival, OS: overall survival) (DOCX 185 kb)

Supplementary Fig. 8 Kaplan-Meier evaluation of the RDBN grading system. There was no sign of significant difference among subgroups (RDBN: Residual disease in breast and nodes) (DOCX 159 kb)

## Data Availability

The datasets generated during and/or analysed during the current study are available from the corresponding author on reasonable request.

## Code Availability

There are no restrictions on the availability of materials, data and code.
