# Supplementary figures and images for: Examination of Tumor Regression Grading Systems in Breast Cancer Patients Who Received Neoadjuvant Therapy
Source: Pathol Oncol Res. 2020 Jul 20;26(4):2747–54. doi: 10.1007/s12253-020-00867-3 (PMC7471177; doi:10.1007/s12253-020-00867-3)

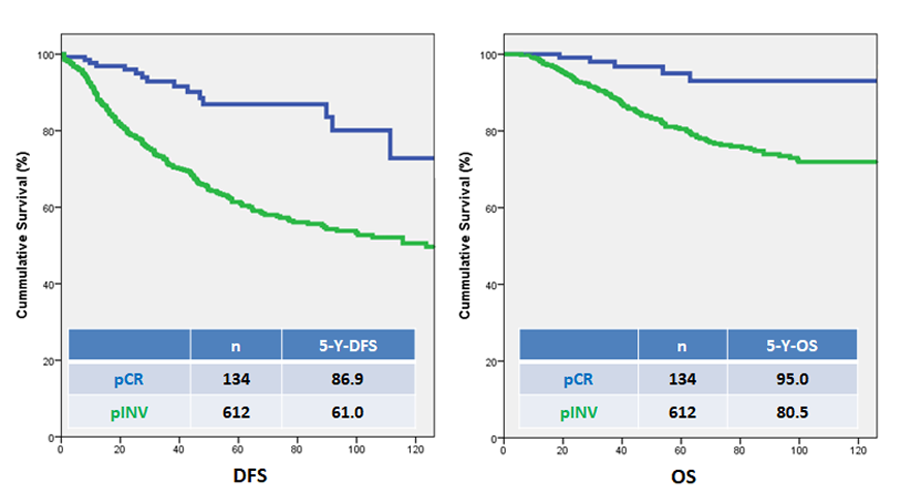

Supplement: Supplementary file 1 — Kaplan-Meier evaluation of the NSABP-B18 response scheme. Significant differences were defined between DFS and OS estimates of pCR vs. residual invasive tumors (pINV) [p < 0.001] (NSABP: National Surgical Adjuvant Breast and Bowel Project, DFS: disease-free survival, OS: overall survival, pCR: pathological complete regression) (DOCX 160 kb) [file 12253_2020_867_MOESM1_ESM.docx]

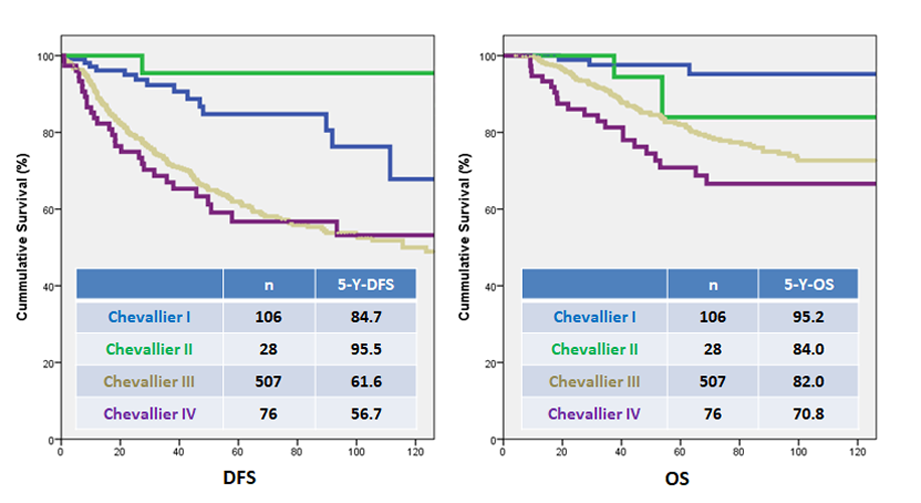

Supplement: Supplementary file 2 — Kaplan-Meier evaluation of the Chevallier grading system. Significant differences were observed between DFS estimates of I vs. III group (p < 0.001); I vs. IV group (p < 0.001); II vs. III group (p < 0.001) and II vs. IV group (p < 0.001). Significant distinction was detected between OS estimates of I vs. III group (p < 0.001); I vs. IV group (p < 0.001) and II vs. IV group (p = 0.05) (DFS: disease-free survival, OS: overall survival) (DOCX 217 kb) [file 12253_2020_867_MOESM2_ESM.docx]

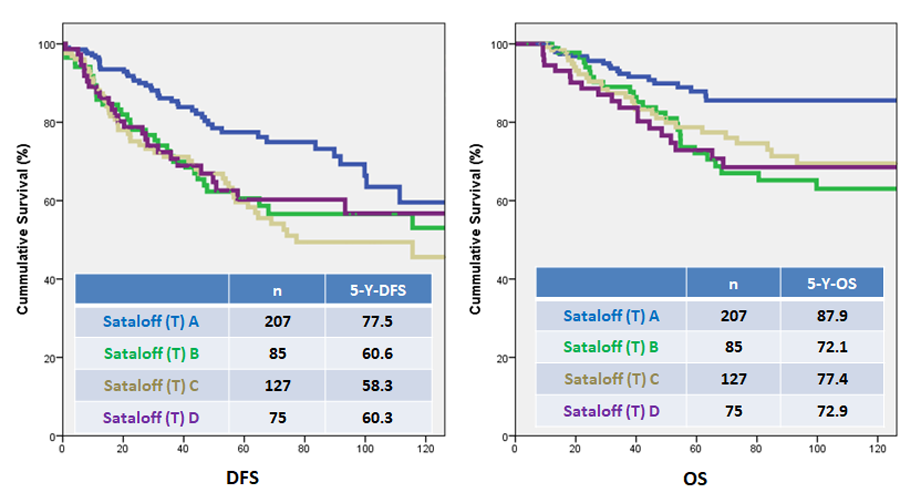

Supplement: Supplementary file 3 — Kaplan-Meier evaluation of the Sataloff (T) grading system. Significant differences were seen between DFS estimates of TA vs. TB (p = 0.005), TA vs. TC (p < 0.001) and TA vs. TD (p = 0.009) along with significant distinction between OS estimates of TA vs. TB (p = 0.005), TA vs. TC (p = 0.016) and TA vs. TD (p = 0.003) (DFS: disease-free survival, OS: overall survival) (DOCX 212 kb) [file 12253_2020_867_MOESM3_ESM.docx]

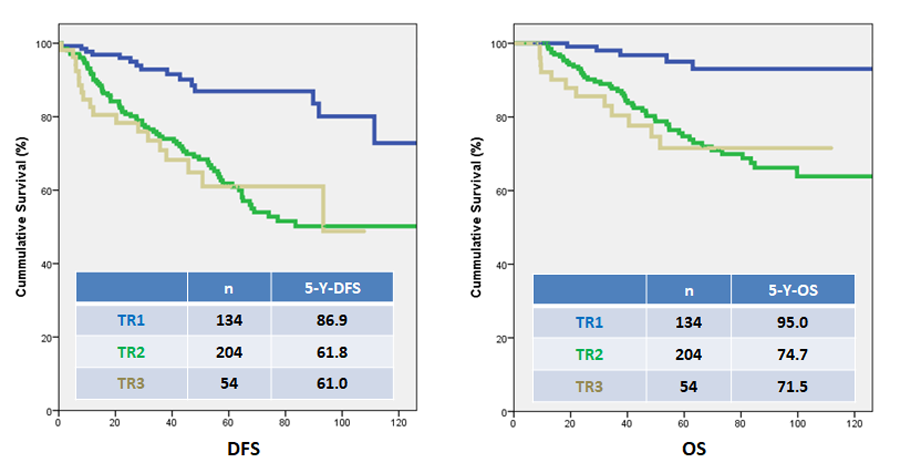

Supplement: Supplementary file 4 — Kaplan-Meier evaluation of the TR grading system. Significant differences were found between DFS and OS estimates of TR1 vs. TR2 (pDFS<0.001; pOS < 0.001) and TR1 vs.TR3 (pDFS<0.001; pOS < 0.001), respectively (DFS: disease-free survival, OS: overall survival) (DOCX 160 kb) [file 12253_2020_867_MOESM4_ESM.docx]

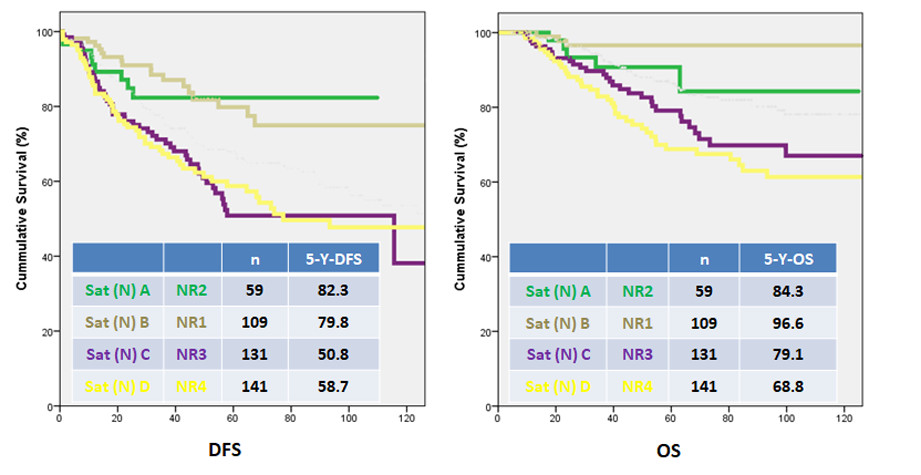

Supplement: Supplementary file 5 — Kaplan-Meier examination of Salatoff (N) and NR grading systems. Significant differences were found between DFS estimates of NR2 vs. NR3 (p = 0.027), NR2 vs. NR4 (p = 0.020), NR1 vs. NR3 (p < 0.001), NR1 vs. NR4 (p < 0.001) and between OS estimates of NR2 vs. NR4 (p = 0.029), NR1 vs. NR3 (p < 0.001) and NR1 vs. NR4 (p < 0.001) (DFS: disease-free survival, OS: overall survival) (DOCX 205 kb) [file 12253_2020_867_MOESM5_ESM.docx]

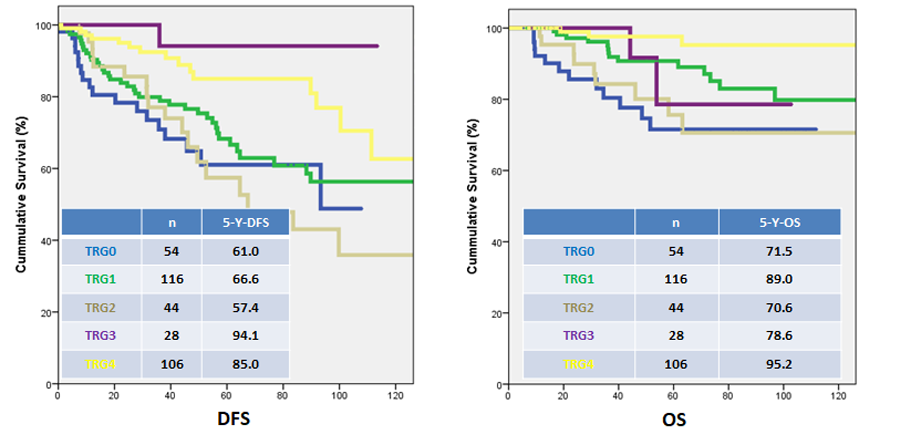

Supplement: Supplementary file 6 — Kaplan-Meier evaluation of the Denkert-Sinn grading system. Significant differences were defined between DFS estimates of TRG3 vs. TRG0 (p = 0.006), TRG3 vs. TRG1 (p = 0.020), TRG3 vs. TRG2 (p = 0.006), TRG4 vs. TRG0 (p < 0.001), TRG4 vs. TRG1 (p = 0.012), TRG4 vs TRG2 (p < 0.001) and between OS estimates of TRG4 vs. TRG0 (p < 0.001), TRG4 vs. TRG1 (p = 0.038), TRG4 vs. TRG2 (p < 0.001) (DFS: disease-free survival, OS: overall survival) (DOCX 176 kb) [file 12253_2020_867_MOESM6_ESM.docx]

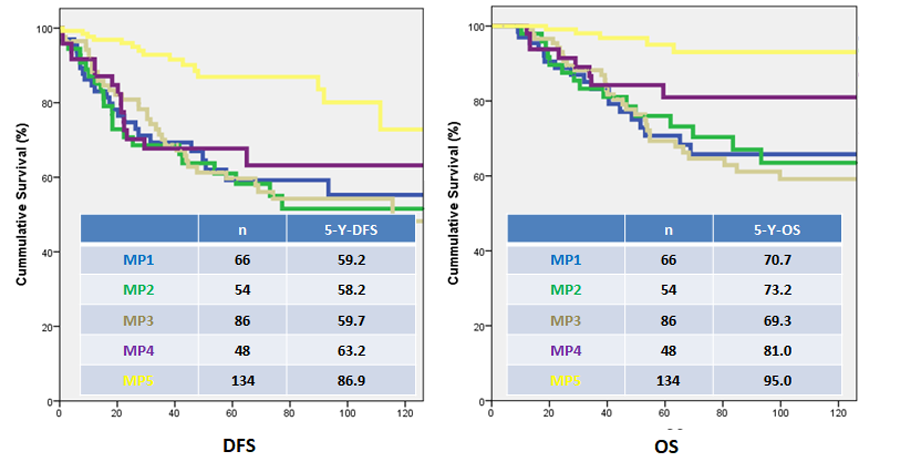

Supplement: Supplementary file 7 — Kaplan-Meier evaluation of the Miller-Payne grading system. The DFS and OS estimates of MP5 group showed significant differences from other groups regarding DFS and OS (p < 0.001) (DFS: disease-free survival, OS: overall survival) (DOCX 185 kb) [file 12253_2020_867_MOESM7_ESM.docx]

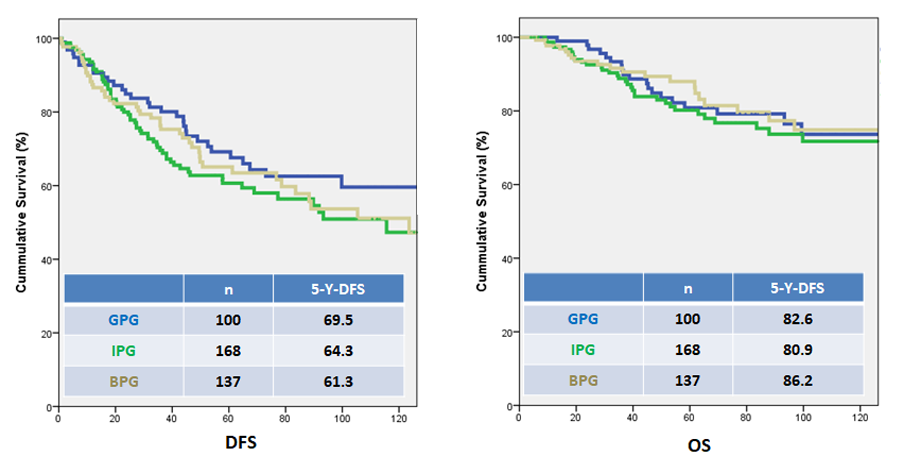

Supplement: Supplementary file 8 — Kaplan-Meier evaluation of the RDBN grading system. There was no sign of significant difference among subgroups (RDBN: Residual disease in breast and nodes) (DOCX 159 kb) [file 12253_2020_867_MOESM8_ESM.docx]
